# Ashwagandha (Withania somnifera) Reverses β-Amyloid_1-42_ Induced Toxicity in Human Neuronal Cells: Implications in HIV-Associated Neurocognitive Disorders (HAND)

**DOI:** 10.1371/journal.pone.0077624

**Published:** 2013-10-16

**Authors:** Kesava Rao Venkata Kurapati, Venkata Subba Rao Atluri, Thangavel Samikkannu, Madhavan P. N. Nair

**Affiliations:** Department of Immunology, Institute of NeuroImmune Pharmacology, Herbert Wertheim College of Medicine, Florida International University, Modesto A. Maidique Campus, Miami, Florida, United States of America; Torrey Pines Institute for Molecular Studies, United States of America

## Abstract

Alzheimer’s disease (AD) is characterized by progressive dysfunction of memory and higher cognitive functions with abnormal accumulation of extracellular amyloid plaques and intracellular neurofibrillary tangles throughout cortical and limbic brain regions. At present no curative treatment is available, and research focuses on drugs for slowing disease progression or providing prophylaxis. Withania somnifera (WS) also known as ‘ashwagandha’ is used widely in Ayurvedic medicine as a nerve tonic and memory enhancer. However, there is a paucity of data on the potential neuroprotective effects of W.somnifera against β-Amyloid (1–42)-induced neuropathogenesis. In the present study, we have tested the neuroprotective effects of methanol:Chloroform (3:1) extract of ashwagandha against β-amyloid induced toxicity and HIV-1_Ba-L_ (clade B) infection using a human neuronal SK-N-MC cell line. Our results showed that β-amyloid induced cytotoxic effects in SK-N-MC cells as shown by decreased cell growth when tested individually. Also, confocal microscopic analysis showed decreased spine density, loss of spines and decreased dendrite diameter, total dendrite and spine area in clade B infected SK-N-MC cells compared to uninfected cells. However, when ashwagandha was added to β-amyloid treated and HIV-1 infected samples, the toxic effects were neutralized. Further, the MTT cell viability assays and the peroxisome proliferator-activated receptor-γ (PPARγ) levels supported these observations indicating the neuroprotective effect of WS root extract against β-amyloid and HIV-1_Ba-L_ (clade B) induced neuro-pathogenesis.

## Introduction

Alzheimer’s disease (AD), is the most common form of senile dementia, affecting more than 15 million people worldwide [1]. With increased life expectancy this number will certainly rise rapidly in the future. AD is characterized by progressive dysfunction of memory and higher cognitive functions associated with memory loss and language deficit which are often accompanied by behavioral and psychological symptoms such as depression, stress, anxiety and mood disturbances [2,3]. The pathological hallmarks are complex and include neuronal degeneration (cholinergic neurons in particular), abnormal neurofibrillary tangles, toxic β-amyloid (AB) plaques, decline of neurochemicals which are essential for neuronal transmission and neuro-inflammation [4-6]. The β-amyloid cytotoxicity to neuronal cells has been identified as one of the major features in AD pathology, but the exact mechanisms involved leading to neurotoxicity still remain an enigma [7]. The transmembrane protein CD33 is a sialic acid-binding immunoglobulin-like lectin that regulates innate immunity but has no known functions in the brain, is considered as a risk factor for Alzheimer’s disease (AD). Very recently an increased expression of CD33 in microglial cells in AD brain was observed [8]. However, the minor allele of the *CD33* SNP rs3865444 confers protection against AD and was associated with reductions in both CD33 expression and insoluble amyloid beta 42 (Aβ42) levels in AD brain [8]. Also, free radical formation and oxidative stress appears to be one of the possible mechanisms involved in AB-induced cytotoxicity [9]. The majority of AD cases are sporadic in nature. However, few familial cases are caused primarily by mutations in three genes namely, amyloid precursor protein (APP), presenilin1 (PS1), and presenilin 2 (PS2) [10]. Neuronal degeneration is also a major feature in HIV infection. A significant increase in brain APP in AIDS, specifically in the axons present in the subcortical white matter tracts have been described by several investigators [11-13]. It has been reported that HIV persists in the brain during HAART therapy and that the local inflammatory responses to HIV in the brain could lead to increased APP production and susceptibility to amyloid deposition [14]. All these observations clearly indicate that β-amyloid accumulation may be a good indicator of early neuronal (axonal) degeneration not only during the development of Alzheimer’s disease but also during HIV induced neuronal degeneration. Recently, good progress has been made in developing the in vitro models for studying the toxic effects of β-amyloid and related peptides in cell cultures, using central nervous system (CNS) neurons or a variety of cell lines of neural origin [15]. 

*Withania somnifera* (L.) Dunal, also known as ‘ashwagandha’ (ASH) in Sanskrit and as ‘Indian ginseng’ is a multipurpose medicinal plant with remarkable increase in recent years in the pharmacological studies, as it has been shown to possess wide spectrum of therapeutic properties such as nerve tonic, memory enhancer, antistress, immunomodulatory and antioxidant properties [16,17]. Withanolide A and withanoside IV from roots help to promote neurite outgrowth in cultured neurons and in rodents injected with Aβ 25–35 [18]. Root extracts from this species have also been shown to significantly reduce the number of hippocampal degenerating cells in the brains of stressed rodents [19] and were neuro-protective in animal models of Parkinson’s disease [20]. A recent study of oral administration of a semi-purified extract of the root of Withania somnifera consisting predominantly of withanolides and withanosides reversed behavioral deficits, plaque pathology, accumulation of β-amyloid peptides (Aβ) and oligomers in the brains of middle-aged and old APP/PS1 Alzheimer’s disease transgenic mice [21]. However, there is a paucity of data on the molecular mechanisms associated with the potential protective effects of W.somnifera root, as used traditionally, against β-amyloid (1–42)-induced cytotoxicity and HIV-1_Ba-L_ (clade B) infection. Accordingly, we hypothesized that ashwagandha may reverse the neuronal toxicity induced by β-Amyloid and HIV-1_Ba-L_ (clade B) infection which may serve as potential therapeutic agent for use in AD and possibly in other HIV related disorders involving memory deficiency. We now report that β-amyloid induced cytotoxic effects in SK-N-MC cells as shown by decreased cell growth when tested individually. Also, confocal microscopic analysis showed decreased spine density, loss of spines and decreased dendrite diameter, total dendrite and spine area in clade B infected SK-N-MC cells compared to uninfected cells. However, when ashwagandha was added to β-amyloid treated and HIV-1 infected samples, the toxic effects were neutralized. Further, the MTT cell viability assays and the peroxisome proliferator-activated receptor-γ (PPARγ) levels supported these observations indicating the neuroprotective effect of WS root extract against β-amyloid and HIV-1_Ba-L_ (clade B) induced neuro-pathogenesis.

## Materials and Methods

### Cell Culture

The effects of β-amyloid and Ashwagandha were tested on the human neuronal cell line, SK-N-MC, obtained from American Type Culture Collection (ATCC) (catalog # HTB-10; Manassas,VA). The cells were grown in T-75 flasks containing Eagle’s minimum essential medium (MEM) (GIBCO) with fetal bovine serum to a final concentration of 10% and 1% antibiotic / antimycotic solution. The cells were maintained in a humidified, 95 % air and 5 % CO_2_ atmosphere incubator at 37° C. 

### Fibrillar β-Amyloid_1-42_ (Aβ_1-42_) or “seed” preparation

Fibrillar **Aβ_1-42_** was prepared as described by Wogulis et al [22]. One mg of **Aβ_1-42_** lyophilized powder (catalog # A9810, SIGMA) was dissolved in 200 µl of water in glass vial and aged for 3 days at 37°C and was diluted with tissue culture medium to the required concentration, before adding to neuronal cultures.

### Extracts of Withania somnifera roots

The dried roots of Withania somnifera were purchased from an authenticated source in Kerala, India. The ground powder (15g) was suspended in 300 ml of solvents (Methanol:Chloroform)(3:1), refluxed for 3 hr. and the supernatant collected. The residue was again suspended in 300 ml of same solvent and refluxed for another 3 hr. and the supernatant collected. Both the supernatants were combined, filtered to remove insoluble material and concentrated to dryness using a rotary vacuum evaporator. The dried extract was solubilized in dimethylsulfoxide (DMSO), aliquoted, stored at -20°C and utilized for all experiments. 

### Analysis and Identification of Methanol: Chloroform (3:1) Fraction

A solution of the extract in dimethylsulfoxide (DMSO) (6 mg /100 µl) was diluted 1:50 in methanol and water (v/v, 1:1). HPLC-MS/MS system consisted of pump (Suryeyor) and an ion trap mass spectrometer equipped with an electrospray ionization (ESI) source (LCQ DECA XP MAX Thermo Finnigan, San Jose, CA, USA). Separations were done on a C18 reversed phase column (5 µm; 4.6 x 25 mm). The column was eluted at a flow rate of 0.5 ml/min with a gradient of water (A) with acetonitrile (B) using the following elution program: 0 min, 95% (A), 5% (B), 0-50 min, a linear gradient to 15% (A), 85% (B). The mass spectrometer was run in the positive ion mode and the operating conditions were as follows: Sheath gas flow: 35 units (Auxiliary gas); capillary temperature: 280° C; spray voltage: 5 KV. For MS / MS experiments, monitored precursor ion precursor isolation width 1µ, relative collision energy 30%. Data processing was performed using Xcalibur software.

### Morphological Characteristics

Morphological assays were carried out as described earlier [23-25]. In brief, approximately 0.1 x 10^6^ SK-N-MC cells obtained from sub-confluent culture flasks were seeded per T-25 flasks in 5 ml complete medium (five flasks per point) or 3 x 10^3^ / 3 ml in 6-well plates (one six well plate per point). Twenty four hours after seeding the cells, the culture medium was removed and serum free medium was added at the same volume. The controls received only solvent and β-amyloid and β-amyloid plus ashwagandha cultures received β-amyloid at a concentration of 5 µM. Different investigators have used different dose levels of β-amyloid depending on the cell type utilized. Michaelis et al [26] used 5 and 10 µM concentration on cortical cell cultures whereas Yankner et al [27] used 20 µM on hippocampal neurons. Kumar et al [28] utilized 0.007-2 µg/mL concentration on PC12 cell line and London et al [29] 0.2, 2.0 and 20 µM on peripheral blood monocytes (PBM). We have standardized the doses required for our studies and used the selected dose for all our experiments. After forty eight hours, ashwagandha was added at 0.15 µg/ml (at this concentration ashwagandha showed growth stimulatory effects) to plain ashwagandha control and β-amyloid plus ashwagandha cultures and media doubled at 10% final serum concentration. For ashwagandha additions, DMSO served as the vehicle to dilute the compound at a final concentration of 0.4% volume per volume and at this concentration has no effect on cell survival. Control cultures received only solvent in the place of test compound. The culture flasks/plates were returned to incubator for up to 3 to 4 days and then washed with PBS solution, fixed with methanol and stained with Giemsa / Sulphorhodamine B (SRB) and pictures were taken. The experiment was repeated three times and depicted for one. 

### MTT Cell Viability Assay

The MTT cell viability assay was carried out by the modified assay as described by Rao et al. [30]. The basic protocol was same as in morphological characteristics but after culture period the 6-well plates were given media change with one ml medium and 100 µl MTT (100 mg MTT / 20 ml PBS) was added for each well and incubated at 37°C for 3 hours. After that, one volume of stop mix solution was added and rocked for about 2 hours, centrifuged and the optical density of the solubilized formazan was determined spectrophotometrically measuring the absorbance at 550 nm. The optical density of formazan in each well is directly proportional to the cell viability and utilized for calculations. 

### Lactate Dehydrogenase Activity (LDH) leakage assay

Cytotoxicity induced by β-amyloid was assessed by lactate dehydrogenase (LDH) leakage into the culture medium. Following exposure to the β-amyloid for 72 hours, the culture medium was aspirated and centrifuged at 3000 rpm for 5 min in order to obtain a cell free supernatant. The activity of LDH in the medium was determined using a commercially available kit from Sigma-Aldrich (Catalog# MAK066) according to the manufacturer’s instructions. 

### Internalization of Aβ _1-42_ by Congo red staining

SK-N-MC cells were grown onto 22 mm x 22 mm glass coverslips at a concentration of 5.0 x 10^3^/ 3 ml in 6-well plates for 48 hours and after that changed to 1 ml of serum free medium. Ashwagandha was added at 0.15 µg/ml to plain ashwagandha control and β-amyloid plus ashwagandha cultures. For ashwagandha additions, DMSO served as the vehicle to dilute the compound at a final concentration of 0.4% volume per volume and at this concentration has no effect on cell survival. Control cultures received only solvent in the place of test compound. Three hours after pre-incubation of cells with WS root extract, β-amyloid and β-amyloid plus ashwagandha cultures received β-amyloid at a concentration of 5 µM. After 16 hours, cells were washed with PBS, fixed in 4% formalin for 15 min at room temperature. Again cells were washed with PBS and then stained with a fresh alkaline solution of 0.5% filtered Congo red (SIGMA-ALDRICH) at room temperature for 5 min. After several washes with deionized water, slides were mounted in glycerol / distilled water (1:1) plus 0.1% sodium azide (SIGMA-ALDRICH) and then observed through a TCS SP2 Confocal Laser Scanning Microscope (Leica Microsystems, Germany) 

### DIL staining and Spine Density Analysis using Confocal Microscopy

SK-N-MC cells were grown onto 22 mm x 50 mm glass coverslips placed in a 100 mm petri-dish at a concentration of 1 x 10^6^ in 8 ml complete medium for 48 hours and after that changed to 8 ml of serum free medium. Ashwagandha was added at 0.15 µg / ml to plain ashwagandha control and β-amyloid plus ashwagandha cultures. For ashwagandha additions, DMSO served as the vehicle to dilute the compound at a final concentration of 0.4% volume per volume and at this concentration has no effect on cell survival. Control cultures received only solvent in the place of test compound. Three hours after pre-incubation of cells with WS root extract, β-amyloid and β-amyloid plus ashwagandha cultures received β-amyloid at a concentration of 5 µM. After another 48 hours, cells were washed with PBS, fixed with 4% formaldehyde in PBS for 30 min at room temperature. DIL staining was performed using a method described previously [31]. The fluorescent membrane tracer, 1, 1’-Dioctadecyl-3, 3,3’,3’-tetramethylindocarbocyanine perchlorate (DIL) at 7.5 mg/ml (in PBS) concentration was directly added onto the fixed cultures and allowed to incubate for 90 min at RT. These stained coverslips were placed overnight at 4° C in petri dishes containing PBS before proceeding for confocal microscopy. Confocal images were obtained using TCS SP2 Confocal Laser Scanning Microscope (Leica Microsystems, Germany) at 488 nm (100%) illusion of an argon-ion laser using 60X oil immersion objectives with high numeric aperture and 2.5X confocal electronic zoom settings to visualize individual cells. Twenty Optical serial sections of 0.14 µm / section (~ 2.8 µm total) through the cells were captured and reconstructed to yield complete ‘‘two dimensional’’ images of individual cells in focus.

### HIV-1 infection of SK-N-MC human neuronal cells

SK-N-MC human neuronal cells were infected with HIV-1 using the previously described protocol [31] with slight modifications. Briefly, SK-N-MC (0.1×10^6^ cells) cells were cultured onto 22 mm x 50 mm glass coverslips placed in a 100 mm petri-dish overnight in 10 ml of medium. In the morning polybrene (25 µg/ 5ml) was added for 6–7 hours and after that cells were infected with 100 ng of HIV-1_Ba-L_ (clade B) (NIH AIDS Reagent Program, Cat. # 510) overnight in respective petri-dishes. Next day morning, cells were washed with PBS to remove unattached virus and replaced with fresh 10 ml medium. Ashwagandha was added at 0.15 µg / ml to plain ashwagandha control and HIV plus ashwagandha cultures and cultured for 72 hours and utilized for confocal microscopy as described above. The supernatant obtained from the used medium was used for the p24 antigen estimation using ELISA kit (ZeptoMetrix Corp. Cat # 0801200). Controls cells (without clade B) were included in the set-up of all experiments.

### Western Blot Analysis

SK-N-MC (1x10^6^) cells were cultured in T-75 flasks in 8 ml complete medium for 48 hours and after that changed to 8 ml of serum free medium. Ashwagandha was added at 0.15 µg / ml to plain ashwagandha control and β-amyloid plus ashwagandha cultures. For ashwagandha additions, DMSO served as the vehicle to dilute the compound at a final concentration of 0.4% volume per volume and at this concentration has no effect on cell survival. Control cultures received only solvent in the place of test compound. Three hours after pre-incubation of cells with WS root extract, β-amyloid and β-amyloid plus ashwagandha cultures received β-amyloid at a concentration of 5 µM. After another 48 hours, cells were washed with PBS solution and were harvested using Trypsin/EDTA solution (ScienceCell Laboratories), cell pellets were collected and lysed using lysis buffer (Pierce, IL) with 1x complete cocktail of protease inhibitors. Total cellular protein in equal quantity was resolved by 4-15% polyacrylamide gel electrophoresis, transferred to a nitrocellulose membrane. The following primary antibodies were used: anti-PPARγ, H-100 and anti-GAPDH (Santa Cruz Biotechnology). Immunoreactive bands were visualized using a chemiluminescence’s Western blotting system according to the manufacturer’s instructions (Amersham).

### Statistical Analysis

The results were expressed as mean ± standard deviation and the significance was evaluated using the Student’s t-test (GraphPad Software, CA, USA). Results giving p ≤ 0.02 were considered significant.

## Results

Chemoprevention has been acknowledged as an important and practical strategy for the management of several disorders [32-34]. From this point of view, we have selected Withania somnifera (WS) also known as Ashwagandha, which is in common use in Indian traditional medicine to promote cognition, including memory and extracted with a mixture of methanol: chloroform (3:1) for testing the beneficial effects on SK-N-MC, a neuronal cell line. This extract showed growth stimulatory effects on SK-N-MC cell line and accordingly was used for all studies.

### LS-MS Analysis

**Figure 1:**
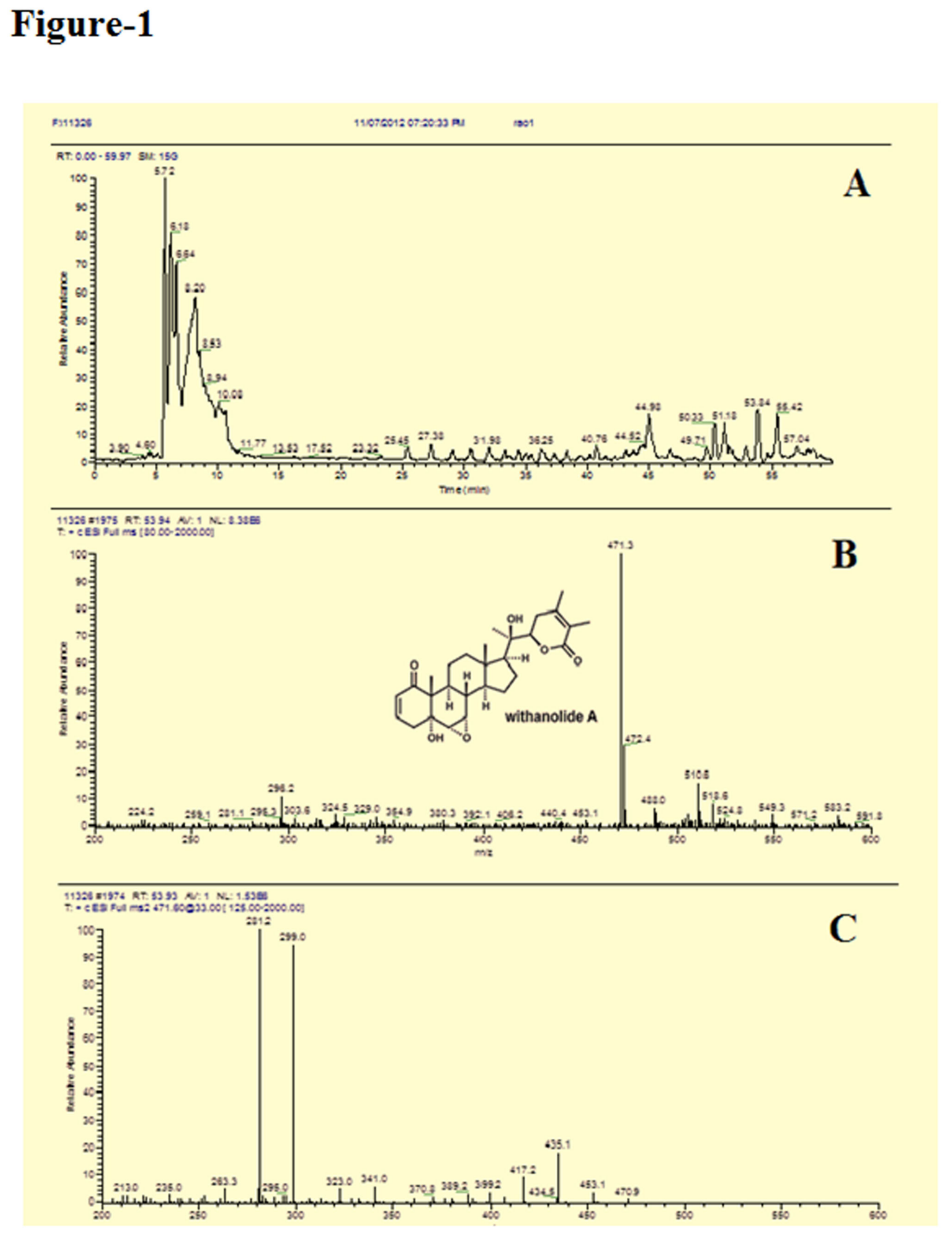
Compounds observed by LC-MS analysis of Methanol:Chloroform (3:1) extract of W.somnifera root. A. HPLC profile showing the main components present. B. UV-Vis and mass spectra of the Withanolide A, identified as Withanolide A by comparison with a reference standard. C. UV-Vis and mass spectra of few other peaks. The structures of these components cannot be ascertained from these data alone and further studies are required.

Positive ion LS-MS analysis of the W.somnifera (WS) extract revealed numerous components (Figure 1). The major constituent Withanolide A was identified against a standard.

### Morphological Characteristics

**Figure 2:**
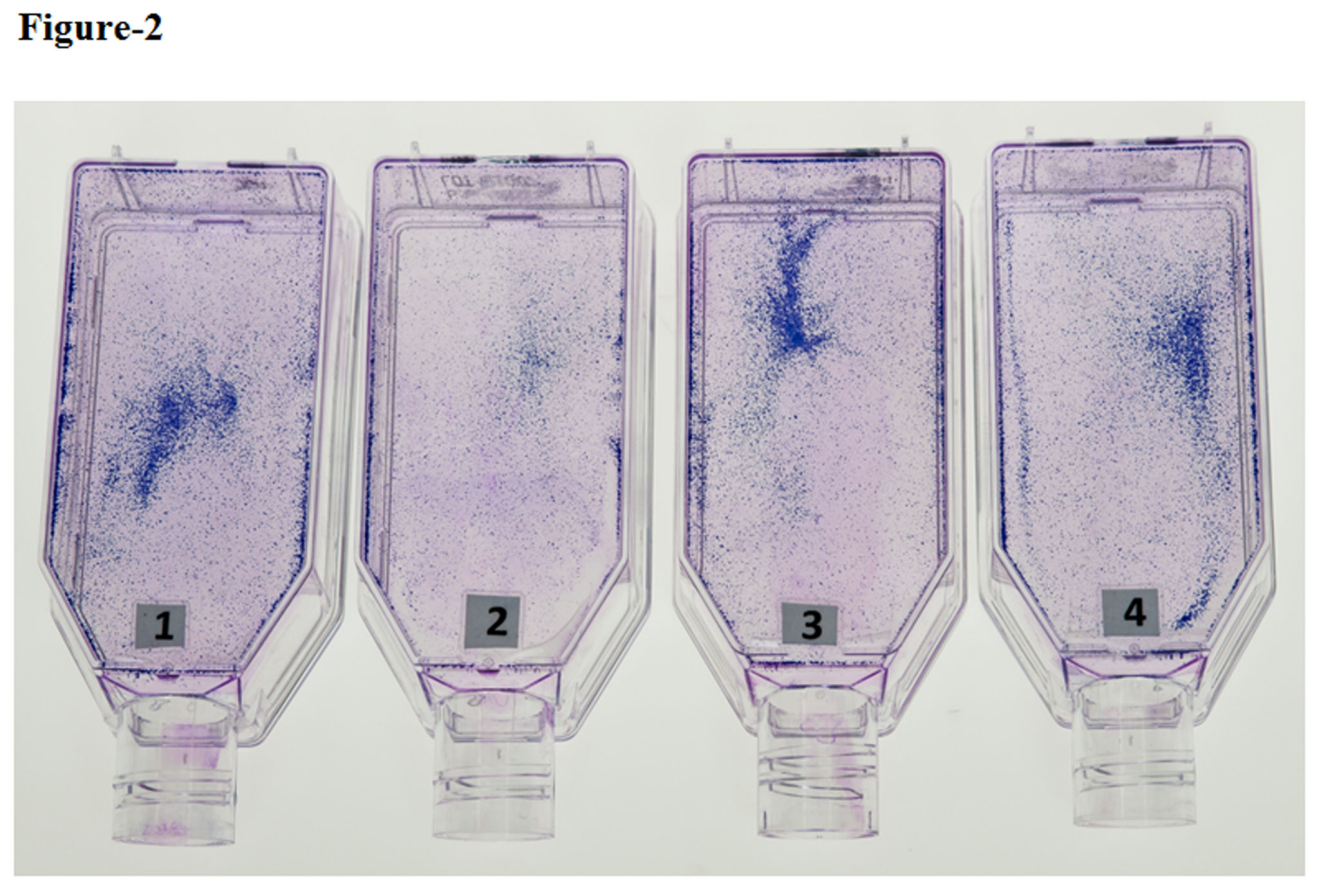
Representative Giemsa stained flasks showing the effect of β-amyloid_1-42,_ Ashwagandha and Ashwagandha plus β-amyloid_1-42_ on SK-N-MC cell line 1. Control, 2. β- Amyloid _1-42_ treated, 3. Ashwagandha treated and 4. Ashwagandha plus β- Amyloid_1-42_ treated.

**Figure 3:**
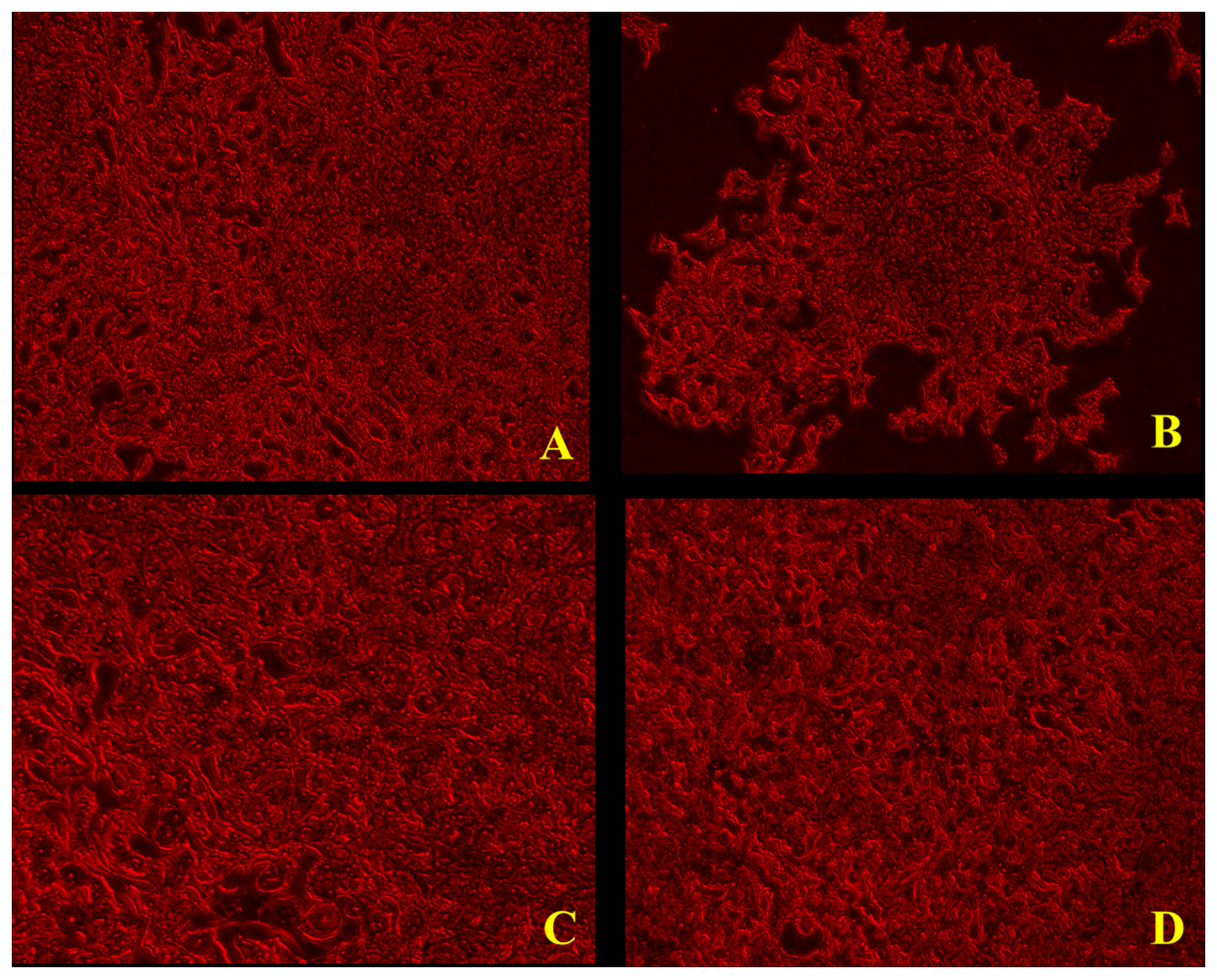
Representative microscopic observations of Sulforhodamine B (SRB) stained cultures showing the effect of β-amyloid_1-42,_ Ashwagandha and Ashwagandha plus β-amyloid_1-42_ on SK-N-MC cell line (X80). A. Control, B. β- Amyloid _1-42_ treated, C. Ashwagandha treated and D. Ashwagandha plus β- Amyloid_1-42_ treated.

In both, Giemsa stained flasks (Figure 2) as well as Sulphorhodamine B (SRB) stained cells (Figure 3), the β-amyloid treated cells showed cytotoxic effects with decreased cell growth compared to plain controls (Figure 2,2 and Figure 3B). However, when ashwagandha was added to β-amyloid treated cultures, the cytotoxic effects of β-amyloid were neutralized and the cells were comparable to plain and ashwagandha treated controls, suggesting the chemopreventive or protective effects of ashwagandha against β-amyloid induced toxicity (Figures 2&3)

### MTT Cell Viability Assay

**Figure 4:**
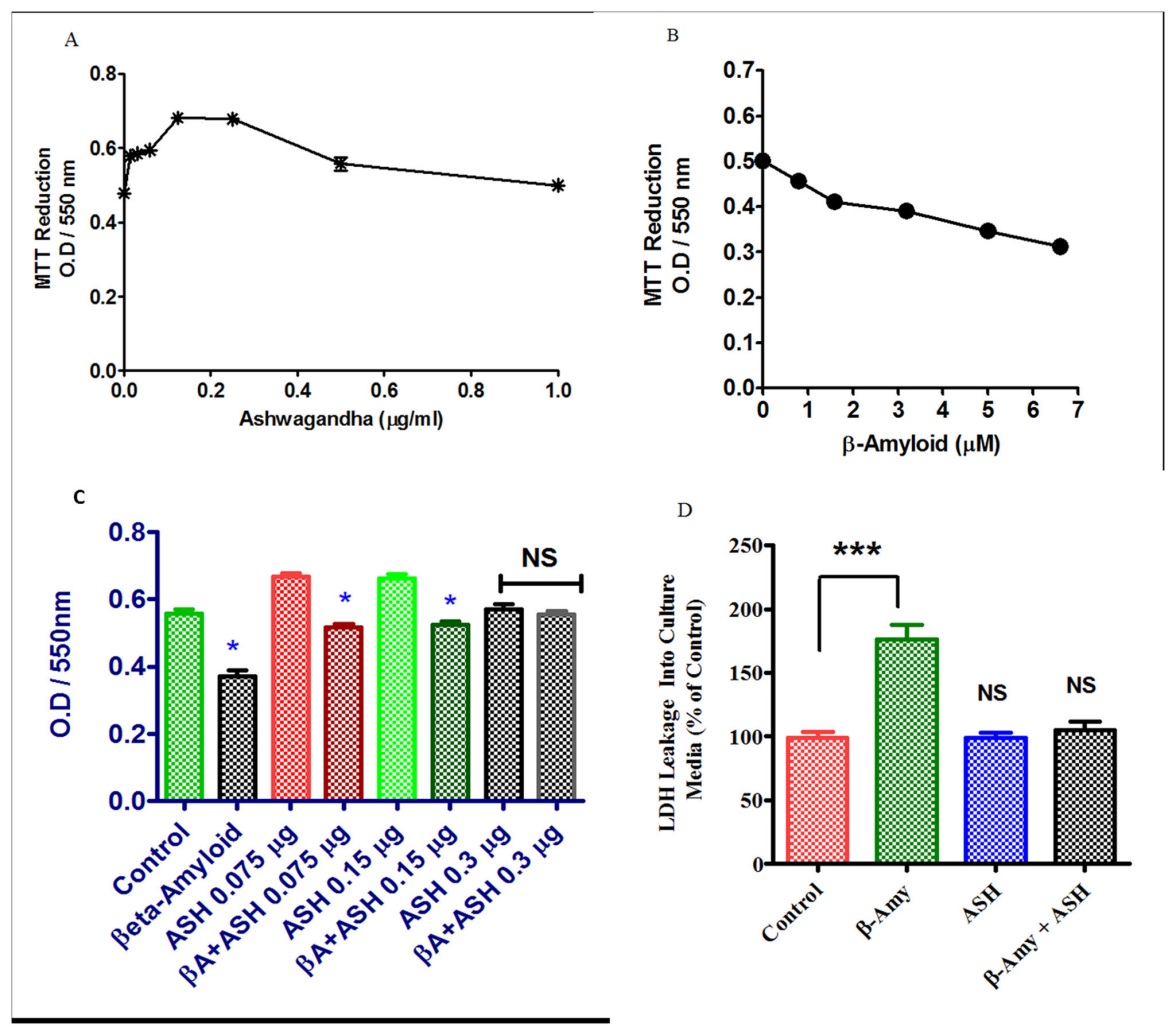
Modulatory effects of Ashwagandha and β- Amyloid_1-42_ on human neuronal cells. A. Dose-response curve of Ashwagandha showing optimal concentrations and B. Dose-response cytotoxic effects of β-amyloid_1-42_ on SK-N-MC cells. Cells were treated with different concentrations of Ashwagandha / β-amyloid_1-42_ and mitochondrial function was determined by the MTT reduction assay as described in the Materials and Methods. C. MTT assay showing the inhibition of cell viability by β- Amyloid_1-42_ (βA) and its reversal by Ashwagandha (ASH) at different concentrations on SK-N-MC cell line D. Effect of β-amyloid_1-42_ on LDH leakage in SK-N-MC cells and its reversal by Ashwagandha. The data are expressed as mean ± SD of four independent experiments. (*) indicates a statistically significant difference compared to controls (p<0.05).

Figure 4A shows the dose-response curve for Ashwagandha on SK-N-MC cells. Ahhwagandha exhibited a significant (p<0.0001) dose-dependent increase in cell viability as reflected by MTT activity from 0.016 to 0.25 µg / ml and there after showed a decline in activity curve. The results of MTT assays also showed that β-amyloid exposure exhibited a significant (p<0.0001) cytotoxicity from 0.825 to 6.6 µM (Figure 4B). Figure 4C shows the histograms of cell viability in control, β-amyloid, ashwagandha and β-amyloid plus ashwagandha treated cultures at different ashwagandha concentrations. The results supported the earlier observations that β-amyloid exerts cytotoxic effects in neuronal cells with decreased cell viability when tested individually. However, when ashwagandha was added to β-amyloid treated cultures, the cytotoxic effects of β-amyloid were neutralized thus showing the beneficial effects. 

### Lactate Dehydrogenase Activity (LDH) leakage assay

The results demonstrated that exposure to β-amyloid to SK-N-MC cells for 72 hours resulted in a significant (p<0.0008) increase in LDH leakage into culture medium indicating cytotoxicity. However, Ashwagandha treatment showed protective effects against the cytotoxicity as the levels of LDH leakage in Ashwagandha plus β-amyloid treated cultures were comparable with controls. (Figure 4D)

### Ashwagandha decreases the internalization of Aβ _1-42_

**Figure 5:**
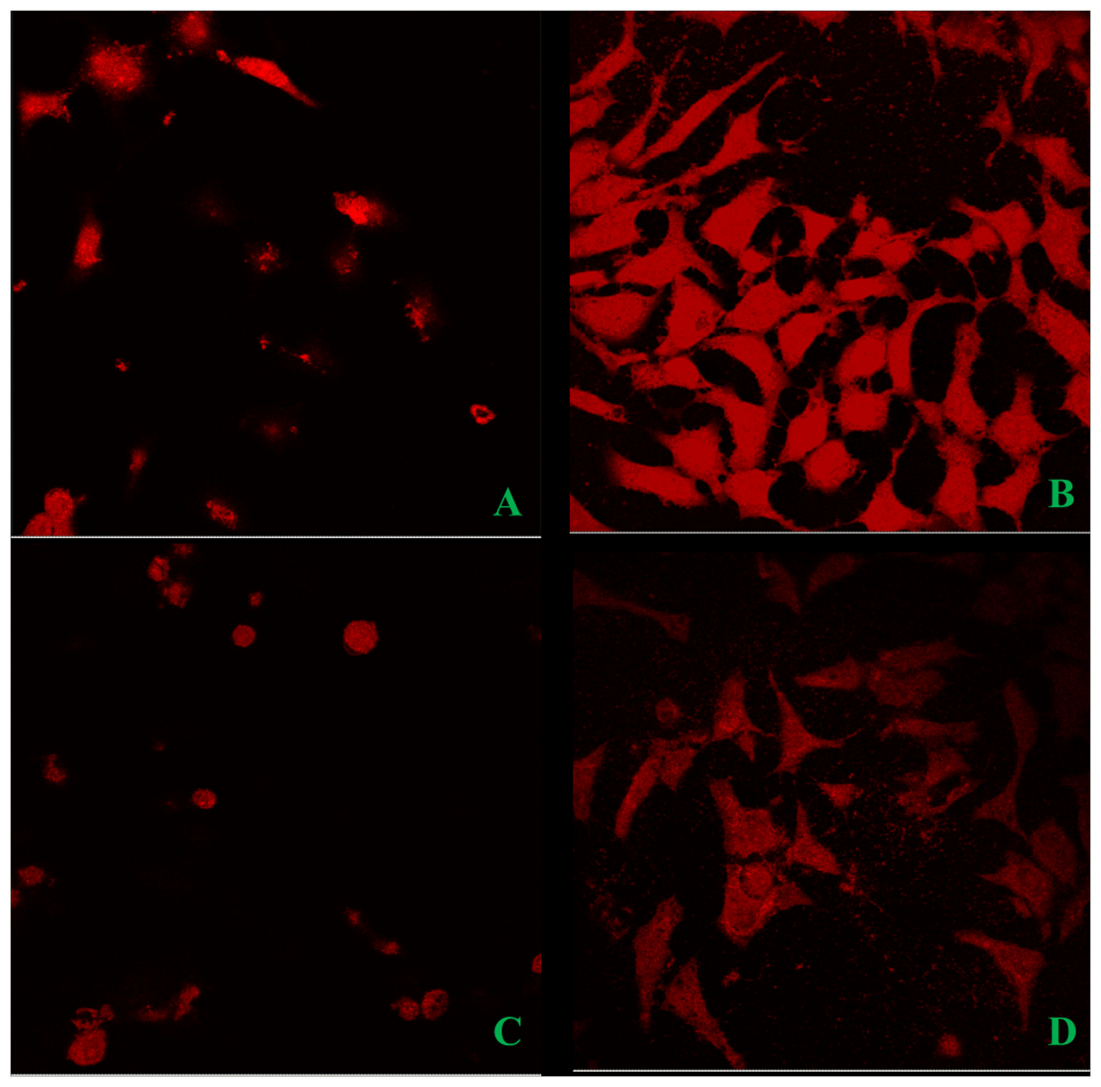
Congo red staining showing the increased internalization in β- Amyloid_1-42_ treated and its reversal by Ashwagandha in SK-N-MC cell line A. Control, B. β- Amyloid _1-42_ treated, C. Ashwagandha treated and D. Ashwagandha plus β- Amyloid_1-42_ treated. β- Amyloid_1-42_ cell internalization was observed by confocal laser microscopy: excitation 488- 543 nm and emission 560 nm; lens 20x / 0.5, 3 x. Images are from one representative experiment of two experiments performed.

In order to understand the effect of ashwagandha on the internalization of β-amyloid_1-42_ in SK-N-MC cells, cells were pre-incubated with extract for three hours and then exposed to β-amyloid_1-42_ for 16 hours. After that cells were stained with Congo red, a metachromatic anionic dye, specific for β-amyloid. As Figure 5 shows, cultures treated with β-amyloid_1-42_ alone showed much more marked internalization of the toxic peptide than occurred when cells were incubated with β-amyloid_1-42_ plus ashwagandha. Plain controls and only ashwagandha treated cultures showed only back ground staining.

### Decreased Spine Density in β-amyloid treated using Confocal Microscopy

**Figure 6:**
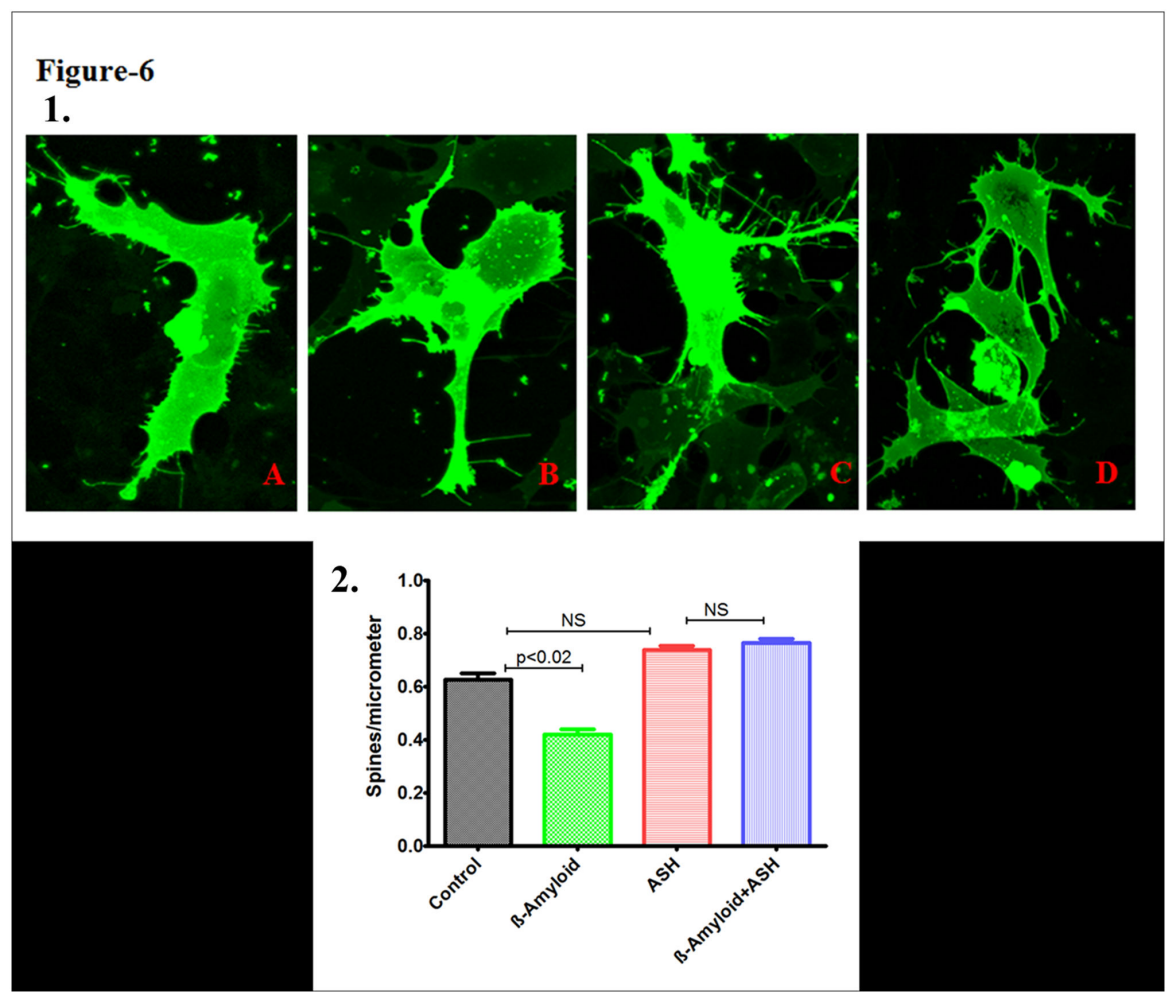
Confocal Images of DIL stained SK-N-MC cells showing the effect of β-amyloid_1-42,_ Ashwagandha and Ashwagandha plus β-amyloid_1-42_. **1**. A. Control, B. β- Amyloid _1-42_ treated, C. Ashwagandha treated and D. Ashwagandha plus β- Amyloid_1-42_ treated. 2. Quantitative analysis showing the decreased spine density in β- Amyloid_1-42_ treated SK-N-MC cell line and its reversal by Ashwagandha (ASH). SK-N-MC cells were grown onto the glass coverslips, DIL stained and observed under confocal microscope. Randomly selected pictures in each group of the cells were captured in confocal microscope. Image J software was used to analyze the spine density, spine area, spine length and number of spines.

Altered cellular morphology and architecture in β-amyloid (Figure 6B) as well as β-amyloid plus ashwagandha (Figure 6D) and control (Figure 6A) and only ashwagandha (Figure 6C) treated SK-N-MC cells were captured using confocal microscopy and morphological changes were analyzed using the established protocol [35]. Treatment of SK-N-MC cells with β-amyloid resulted in significant decrease in spine density (p < 0.02), spine area, spine length and number of spines (B) compared with untreated control cells. In ashwagandha and ashwagandha plus β-amyloid treated cells no reduction in spine density, spine area, spine length and number of spines was observed compared to untreated control (B). 

### Decreased Spine Density in HIV-1 treated using Confocal Microscopy

**Figure 7:**
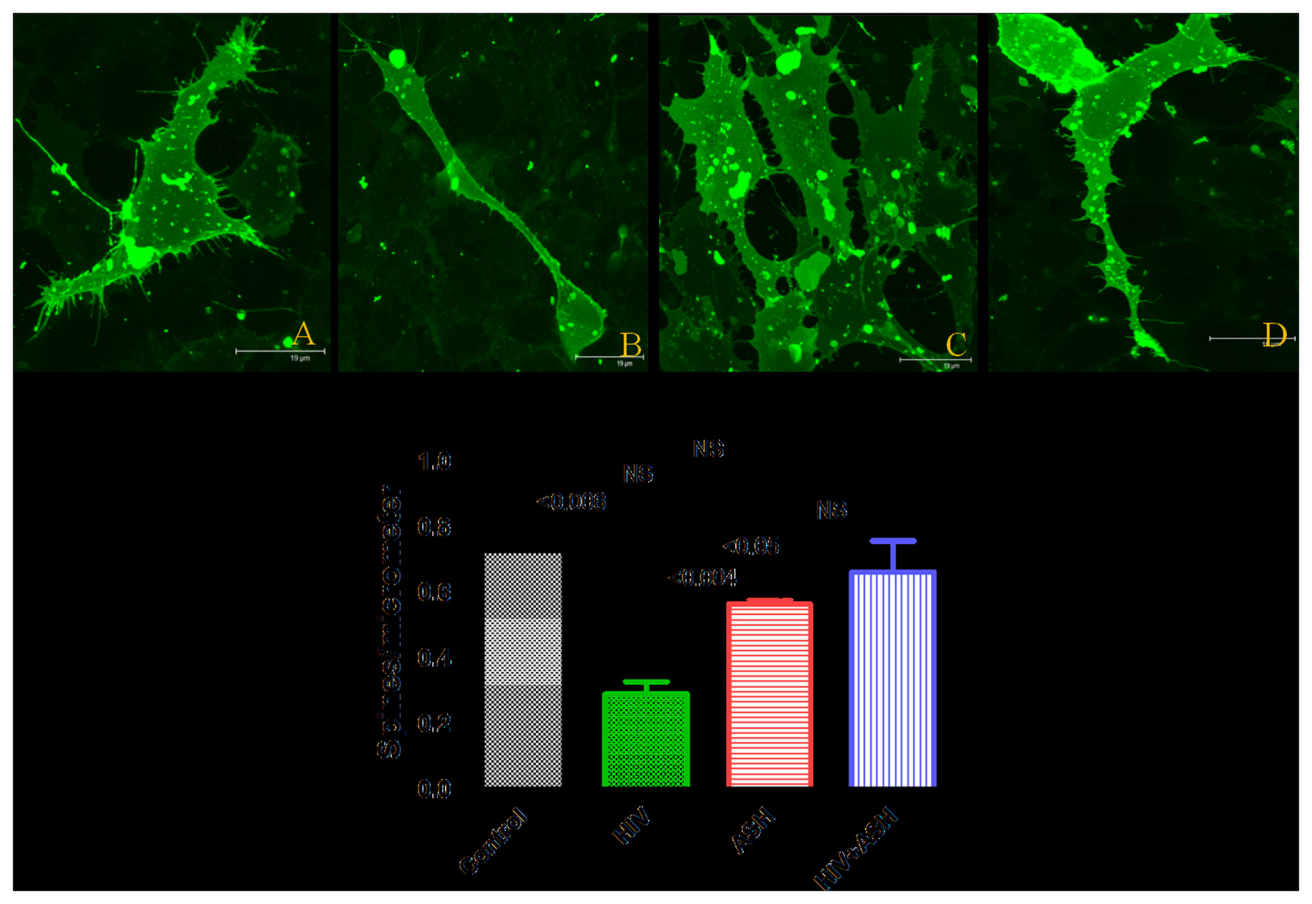
Confocal Images of DIL stained SK-N-MC cells showing the effect of HIV-1_Ba-L_ (clade B), Ashwagandha and Ashwagandha plus HIV-1_Ba-L_ (clade B). 1. A. Control, B. HIV-1_Ba-L_ (clade B) treated, C. Ashwagandha treated and D. Ashwagandha plus HIV-1_Ba-L_ (clade B) treated. 2. Quantitative analysis showing the decreased spine density in HIV-1_Ba-L_ (clade B) treated SK-N-MC cell line and its reversal by Ashwagandha (ASH). SK-N-MC cells were grown onto the glass coverslips, DIL stained and observed under confocal microscope. Randomly selected pictures in each group of the cells were captured in confocal microscope. Image J software was used to analyze the spine density, spine area, spine length and number of spines.

Changes in spine density and dendrite morphology in HIV-1 infected (Figure 7B) as well as HIV-1 plus ashwagandha (Figure 7D) and control (Figure 7A) and only ashwagandha (Figure 7C) treated SK-N-MC cells were captured using confocal microscopy and morphological changes were analyzed using the established protocol [35]. The infection of SK-N-MC cells with HIV-1 resulted in significant decrease in spine density (p < 0.05), spine area, spine length and number of spines (B) compared with untreated control cells. In ashwagandha plus HIV-1 treated cells no reduction in spine density, spine area, spine length and number of spines was observed compared to untreated control (B). 

### Decreased PPARγ levels in β-amyloid treated cultures by Western Blot Analysis

**Figure 8:**
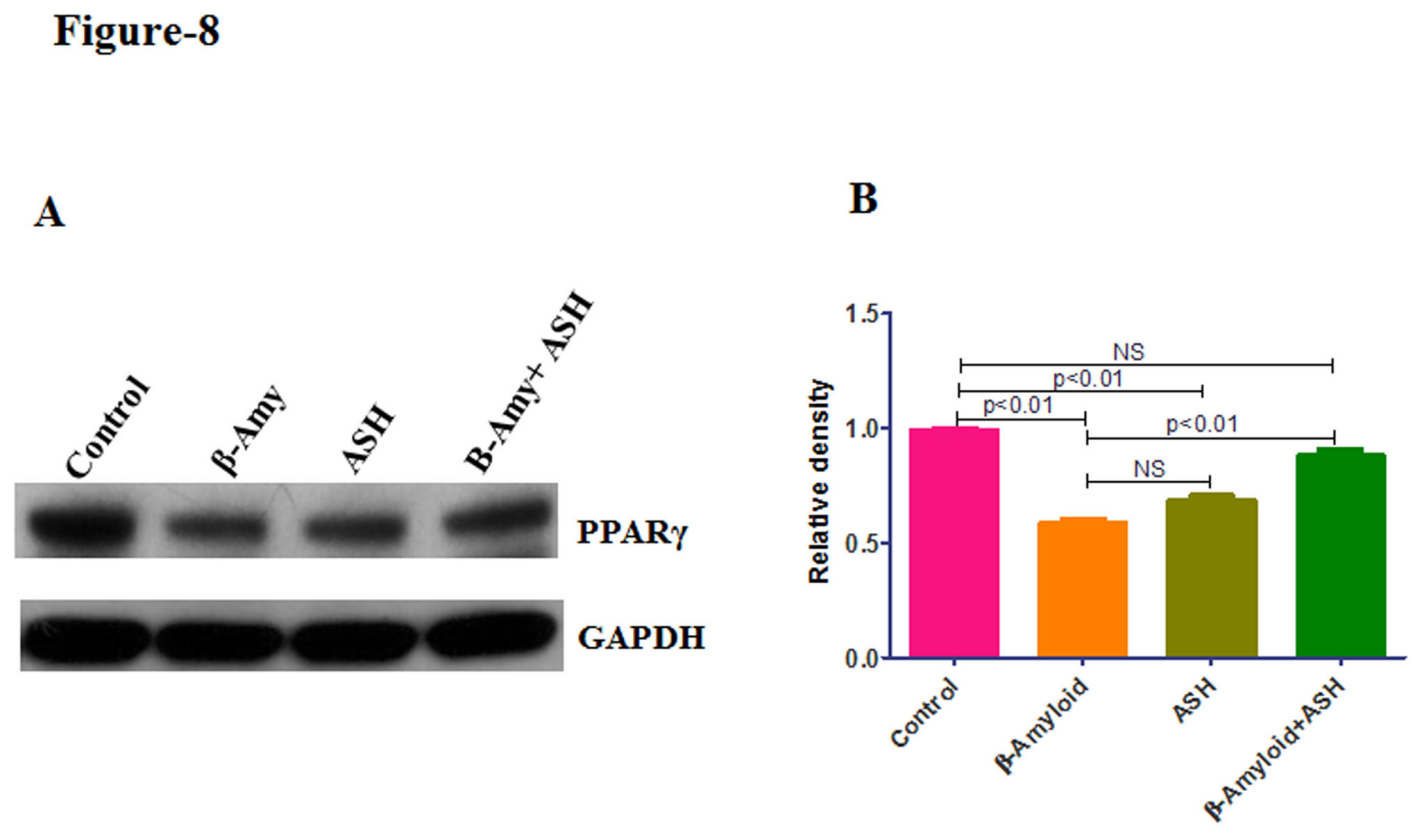
Western blotting analysis showing the decreased PPARγ protein levels in β-amyloid treated and its reversal by Ashwagandha in SK-N-MC neuronal cells. (A) Cell lysates were separated in 4% to 15% linear gradient SDS-PAGE gels and were probed against the respective antibodies. GAPDH was used as the loading control. (B) Quantitative analysis showing the decreased PPARγ protein levels in β- Amyloid_1-42_ treated cultures. ASH - Ashwagandha; β-amy - β-amyloid. The gel shown is a representative for three experiments.

The peroxisome proliferator-activated receptor-γ (PPARγ) is implicated in numerous diseases including Alzheimer’s disease, obesity, diabetes, atherosclerosis and cancer. Accordingly, in order to understand the role of peroxisome proliferator-activated receptors (PPARs) during the exposure of β-amyloid as well as in the combination of β-amyloid plus Ashwagandha, Western blotting analysis was carried out in cell lysates. Treatment of SK-N-MC cells with β-amyloid resulted in decreased PPARγ protein level (p < 0.01) (Figure 8) compared with untreated control cells. In ashwagandha plus β-amyloid treated cells no significant reduction (NS) in protein level was observed compared to untreated control (Figure 8). However, also a relatively slight decrease in PPARγ level was observed in plain Ashwagandha treated cultures. Further studies are required to understand the biological significance of this observation. 

## Discussion

Considerable attention has been focused on the accumulation of β-amyloid peptide (Aβ) within the brain as a major etiologic factor in the pathogenesis of Alzheimer disease (AD). The commonly used drugs for AD like memantine, is a N-methyl-D-aspartic acid (NMDA) receptor antagonist, which may restore the functions of damaged neurons through reducing abnormal excitatory signals via the modulation of NMDA receptor activity [36] and cholinesterase (AChE) inhibitors suppress the enzymatic hydrolysis of neurotransmitter acetylcholine, thus maintain a higher acetylcholine concentration in the neuronal synapse [37], provide a symptomatic relief but no cure for the disease. At present, there is no curative treatment available for AD and the approved medications are used only for slowing the disease progression or providing prophylaxis. Numerous studies over the past two decades indicated that Withania somnifera (WS) has anti-inflammatory, anti-tumor, anti-stress, anti-oxidant, mind boosting and rejuvenating properties [38-40]. Studies also showed that extracts of WS root promote dendrite formation in human neuroblastoma cells in vitro in a dose-dependent manner [41,42]. In the present study WS root extract showed growth stimulatory effects at relatively lower concentrations on SK-N-MC, a neuronal cell line (Figure 4A). Accordingly, this extract was utilized for identification of the components present as well as for other studies. HPLC and mass spectra analysis showed the presence of withanolide A as the main withanolide in the extract (Figure 1). Withanolides are known to be responsible for the multiple medicinal properties of Ashwagandha [43]. Structurally, withanolides consist of a steroid backbone bound to a lactone and have resemblance in their structure and action to ginsenosides, the active constituents of Asian ginseng, Panax ginseng [44]. Besides withanolide A, the presence of different other components were also observed in this extract. Further studies are required to identify these components and their biological significance. 

In both, Giemsa stained flasks (Figure 2) as well as Sulphorhodamine B (SRB) stained cells (Figure 3), the β-amyloid treated cells showed cytotoxic effects with decreased cell growth compared to plain controls (Figure 2,2 and Figure 3B). However, when ashwagandha was added to β-amyloid treated cultures, the cytotoxic effects of β-amyloid were neutralized and the cells were comparable to plain and ashwagandha treated controls, suggesting the chemopreventive or protective effects of ashwagandha against β-amyloid induced toxicity (Figures 2&3). The cell viability was assessed by the 3-(4, 5-dimethylthiazol-2-yl)-2, 5-diphenyltetrazolium bromide (MTT) assay and same pattern was observed (Figure 4C). Previous work showed that β-amyloid is toxic to primary neurons [27,45,46]. Also, It has been reported earlier that exposure of cultured neurons to β-amyloid induces degeneration and cell death through an apoptotic pathway and suggests that activation of an apoptotic pathway may contribute to the neuronal loss associated with AD [47,48]. It is possible that similar mechanisms may be responsible for the cell degeneration observed in the present study during the exposure of β-amyloid (Figure 4B). In the present study, our data shows that ashwagandha prevented this cellular degeneration. Some studies suggest that one pathway of β-amyloid induced cytotoxicity could be mediated by free radicals and oxidative stress [28,49-53]. It is known that ashwagandha has antioxidant and free radical scavenger activity and this could inhibit β-amyloid induced cellular degeneration [54]. Also, ashwagandha inhibits acetylcholinesterase activity and thus has potential to modulate cholinergic function [55-57] or may be connected to clearing effect associated with the degradation of β-amyloid by many proteases, including neprilysin, endothelin-converting enzyme, angiotensin-converting enzyme, plasminogen activator and matrix metalloproteinase-9 [58-62]. 

Accumulating evidence from different clinical studies, transgenic models as well as in vitro studies suggests that intraneuronal accumulation of β-amyloid is an early event and plays an important role in the pathogenesis of AD [63,64]. Further, extracellular addition of β-amyloid to neuronal cells in culture is known to induce the uptake of β-amyloid and its localization to the nucleus [65-68]. In the present study, cultures treated with β-amyloid_1-42_ alone showed much more marked internalization of the toxic peptide compared to cultures treated with β-amyloid_1-42_ plus ashwagandha (Figure 5). 

Neuronal spines are tiny protrusions along cells, which constitute major postsynaptic sites for excitatory synaptic transmission. Alterations in spine density and the formation of new synapses are activity dependent processes that provide a basis for memory formation and function. A loss or alteration of these structures has been described in patients with neurodegenerative disorders such as Alzheimer’s disease [69]. In the present study of confocal microscopy, treatment of SK-N-MC cells with β-amyloid and HIV-1 infection resulted in decrease of spine density, spine area, spine length and number of spines (Figures 6&7) compared with untreated control cells which may negatively affect the synaptic plasticity in neuronal cells. However, in β-amyloid plus ashwagandha as well as HIV-1 plus ashwagandha treated cells no reduction in spine density, spine area, spine length and number of spines was observed compared to untreated control (Figures 6&7) indicating the protective and reversal effect of ashwagandha on spine density. 

Alzheimer’s disease is characterized by disruption of β-amyloid homeostasis, resulting in the accumulation β-amyloid within the brain. The peroxisome proliferator-activated receptor-γ (PPARγ) is a ligand-activated nuclear receptor and its activation is associated with clearance of β-amyloid and ameliorating the pathologic and behavioral deficits in AD [70]. In general, β-amyloid deposition elicits a vigorous “M1” microglia-mediated inflammatory response contributing to disease progression [71-73].. However, activation of PPARγ is associated with the alteration of macrophages and microglia into “M2” state linked with the suppression of inflammation and promotion of β-amyloid phagocytosis and tissue repair [74,75]. In the present study treatment of SK-N-MC cells with β-amyloid resulted in the decreased PPARγ protein levels (p < 0.01) (Figure 8) compared with untreated control cells. However, in ashwagandha plus β-amyloid treated cells no significant reduction in protein levels was observed compared to untreated control (Figure 8). It is possible that in cells treated with only β-amyloid, decreased levels of PPARγ protein levels may contribute to the cytotoxic properties of β-amyloid observed. However, since there is no reduction of PPARγ protein levels in Ashwagandha plus β-amyloid treated cells, the cytotoxic properties of β-amyloid were neutralized probably due to clearance of added β-amyloid and further studies are required to understand the mechanisms. Further, this data supports a possible mechanistic link between PPARγ and amyloid clearance due to Ashwagandha and support the therapeutic use of ashwagandha against AD.

Even though, the mechanisms of AD are still not completely understood, it is believed that excessive accumulation of β-amyloid through abnormal β-amyloid precursor protein (APP) and β-amyloid metabolism are key events in the pathogenesis of AD. Thus, strategies targeting β-amyloid metabolism and APP processing are of immense help for the treatment and prevention of AD. Here we have demonstrated that WS extract reverses the β-amyloid and HIV-1 induced neuronal toxicity in SK-N-MC cells and may serve as potential therapeutic agent for use in AD and possibly in other HIV related disorders involving memory deficiency. WS extract used in the present study is known to contain several compounds besides withanolide A. However, it is reasonable to expect that appropriate combinations of multiple chemopreventive components might provide greater efficacy than the administration of individual component. It is unlikely that chemoprevention of AD can be achieved by a single agent. Accordingly, there is need to develop a mixed cocktail approach with multiple herbal ingredients that act in a concerted way and produce multiple cellular effects with further enhancement of the efficacy in a positive manner for the effective management of AD. Studies are in progress from this point of view.

## Conclusions

In summary, this study demonstrated that Methanol:Chloroform (3:1) extract prepared from the dried roots of W. somnifera and subjected to LC-MS analysis showed the presence of alkaloids and steroidal lactones, the prominent being Withanolide A and was neuroprotective against β-amyloid-induced cytotoxicity and HIV-1 infection. The MTT cell viability assays and confocal studies supported the findings confirming the chemopreventive or protective effects of ashwagandha against β-amyloid induced toxicity and HIV-1 infection. These results further established that neuroprotective properties of the WS root extract observed in the present study may provide some explanation for the ethnopharmacological uses of WS in traditional medicine for cognitive and other HIV associated neurodegenerative disorders. 
